# Morphological and Phylogenetic Analyses Reveal Dictyostelids (Cellular Slime Molds) Colonizing the Ascocarp of *Morchella*

**DOI:** 10.3390/jof10100678

**Published:** 2024-09-28

**Authors:** Wen-Shu Hu, Lin-Lin Jiang, Pu Liu, Xiao-Yan Zhang, Wei Wei, Xi-Hui Du

**Affiliations:** 1 College of Life Sciences, Chongqing Normal University, Chongqing 401331, China; a1292370799@outlook.com (W.-S.H.); jianglinlin1039@163.com (L.-L.J.); xyzhang18@163.com (X.-Y.Z.); weiwei_cqnu@163.com (W.W.); 2 Engineering Research Center of Chinese Ministry of Education for Edible and Medicinal Fungi, Jilin Agricultural University, Changchun 130118, China; pul@jlau.edu.cn

**Keywords:** *Dictyostelium*, 18S rRNA, true morel, multi-gene, distribution, sorocarp

## Abstract

*Morchella* spp. (true morels) are precious edible mushrooms consumed around the world, with a delicious taste, rich nutritional value, and unique healthcare effects. Various fungi and bacteria have been reported to colonize the ascocarps of *Morchella*, damaging their fruiting bodies and leading to serious economic losses in cultivation. The species identification of these colonizing organisms is crucial for understanding their colonization mechanisms on morels. Slime molds, which have characteristics of both “fungi” and “animals”, can occasionally colonize crops and edible fungi. However, there have been no reports of dictyostelid cellular slime molds (dictyostelids) colonizing plants and fungi to date. In this study, we discovered that dictyostelids colonized the surface of one wild ascoma of *Morchella* in the forest of Chongqing, China, with the tissues being black and rotten. Macro- and micro-morphological observations, along with molecular phylogenetic analyses, identified the specimens investigated in this study as *Dictyostelium implicatum* and *Morchella* sp. *Mel*-21. The results provide new knowledge of dictyostelid colonization on organisms and contribute to the diversity of species colonizing true morels. Moreover, this is also the first report of dictyostelids distributed in Chongqing, China. This study enhances our insights into the life history and potential ecological significance of dictyostelids and updates their distribution area in China. Further research will be conducted to uncover the mechanisms behind the colonization observed in this study.

## 1. Introduction

True morels (*Morchella* spp., phylum Ascomycota), a group of the world’s most prized edible and medicinal mushrooms, are of very important economic and scientific value [1]. They are rich in protein, carbohydrate compounds, vitamins, minerals, and other nutrients [2], which have many health benefits, and abundant microorganisms are present on the fruiting bodies [3,4]. Due to the high demand for true morels and their increasing economic importance, morel cultivation has been a global research focus for more than 100 years [5,6]. In recent years, the outdoor cultivation of morels has been successful and greatly expanded in China [6,7]. However, the occurrence of fungi and bacteria colonizing the fruiting bodies of *Morchella* at cultivation sites has been increasingly and commonly reported [8,9,10,11,12,13,14,15] and causes the development of white plaques, dark-black lesions, wrinkled and rotten apothecia, and even perforation symptoms [16,17,18,19,20,21], resulting in decreased harvest yields, declined commodity quality, and reduced final profits [6,22,23,24,25].

Among these harmful organisms colonizing the ascomata of *Morchella*, *Pseudodiploospora longispora* (Matsush.) Jing Z. Sun, X.Z. Liu & H.W. Liu [17,18,26,27] can colonize both the caps and stipes of true morels and are recognized as serious pathogens, which produce numerous conidia spreading rapidly around the cultivation areas, resulting in up to 80% of morel yield losses every year [12]. The *Fusarium incarnatum–F. equiseti* species complex [16] is a group of fungal pathogens distributed worldwide that mainly colonize the stipes of *Morchella importuna* M. Kuo, O’Donnell & T.J. Volk and develop spindle, dark brown, sunken patches with sparse white hyphae on their surfaces. Similar symptoms have also been reported in *Morchella sextelata* M. Kuo due to the colonization of *Clonostachys solani* (Harting) Schroers & W. Gams [21]. Additionally, *Alternaria alternata* (Fr.) Keissl., an opportunistic pathogen noted in economically important fruit crops [9], was found to invade the hymenia of *M. importuna*, resulting in halted fruiting body growth and abnormal morphology [13]. Furthermore, *Purpureocillium lilacinum* (Thom) Luangsa-ard, Houbraken, Hywel-Jones & Samson has been observed to colonize the ascocarps of *Morchella rufobrunnea* Guzmán & F. Tapia [10], while *Trichoderma atroviride* P. Karst [8]., *Pseudomonas chlororaphis* subsp. *aureofaciens* Peix and *Bacillus subtilis* (Ehrenberg) Cohn [15], and *Penicillium raperi* G. Sm. [14] have been documented as pathogens colonizing *M. sextelata.* Unstable environmental conditions provide opportunities for bacteria [15] and fungi [28] to proliferate and colonize morels, damaging their fruiting bodies and causing various diseases [6,21,29]. However, there have been no reports that protists can colonize *Morchella*.

Slime molds, characterized by features of “fungi” and “animals” during their life cycle [30], include endoparasitic slime molds (Phytomyxea), acrasid cellular slime molds (acrasids), dictyostelid cellular slime molds (dictyostelids), plasmodial slime molds (Myxogastrea), and other heterotypic slime molds [31,32,33,34,35]. Most slime molds are saprophytic without significant economic value, and only Myxogastrea can colonize crops and edible fungi in the form of plasmodia or sporangia, affecting the growth of crops and fungi and even causing severe decay and death [36,37,38,39]. For example, *Polymyxa graminis* Ledingham, as a lower eukaryote, obligatorily colonizes plant roots and transmits nine kinds of wheat viruses, specifically *Bymovirus* sp. and *Furovirus* sp. [40]. *Plasmodiophora brassicae* Woronin can colonize plants and damage the roots of most cruciferous plants [41]. In addition, *Stemonitis splendens* Rostaf [42]., *Physarella oblonga* (Berk. & M.A. Curtis) Morgan [43], and *Stemonaria longa* (Peck) Nann.-Bremek., Y. Yamam. & R. Sharma [44] have also been reported to colonize mushrooms. Dictyostelid cellular slime molds (dictyostelids) predominantly inhabit the soil and leaf litter layer, along with animal dung, where they feed mostly on bacteria [45,46,47,48]. Because of their crucial evolutionary status, unique life cycle, and significant interactions with the environment and human health, dictyostelids have become model organisms with significant research value in biological characters, genetics, and applications [49,50]. However, to our knowledge, there have been no reports of dictyostelids colonizing plants or fungi.

In this study, we found that dictyostelids colonized the surface of one wild *Morchella* ascoma in a forest in Chongqing, China, with the tissue observed to be black and rotten. The *Morchella* sample was identified to be *Morchella* sp. *Mel*-21, and the dictyostelids were recognized as *Dictyostelium implicatum* H. Hagiw. based on morphological and molecular phylogenetic studies. Our results contribute to expanding the knowledge about species colonizing *Morchella*, especially wild ascomata, and offer novel perspectives on the potential ecological significance of dictyostelids as well as their distribution in China.

## 2. Materials and Methods

### 2.1. Specimens

The specimens of *Morchella* and dictyostelids were collected from Chongqing, China, in March 2023, then dried with silica gel, and finally deposited in Chongqing Normal University, Chongqing, China. The strain numbers of *Morchella* and dictyostelids are FCNU1120 and H1054, respectively.

### 2.2. Morphological Study of Dictyostelids

#### 2.2.1. Macroscopic Morphological Observation

The macroscopic morphological characteristics of the dictyostelids were observed under a stereo microscope (Leica S9 Series, Shanghai, China), including the size and morphology of aggregations and pseudoplasmodia, the length of sorocarps, the color and branching pattern of sorophores, and the color of sori.

#### 2.2.2. Microscopic Morphological Observation

Several intact and complete dictyostelids were selected and placed on a microscope slide. Before the morphological observation, the specimens were stained with 1% aqueous Congo red solution. Microscopic features were observed using an Optec BK-FL light microscope (Optec, Chongqing, China), including the spore shape and size, the presence of polar granules, macrocysts and microcysts, the width of the sorophores, the number of stalk cell columns, and the characteristics of the top and base of the sorophores. Then, images were captured with an Optec CCD TP510 digital camera (Optec, Chongqing, China) and processed using Adobe Photoshop CC 2019 v.20.0.4.

### 2.3. DNA Extraction, Sequencing, and Phylogenetic Analyses

Under the stereo microscope, ten sorocarps of dictyostelids growing on the cap surface of a single *Morchella* ascoma were randomly selected and transferred to a clean centrifuge tube, and a few tissues from the stipe of *Morchella* where no dictyostelids were observed to colonize were placed in another centrifuge tube. Methods for genomic DNA extraction and Sanger sequencing followed Du et al. [51]. The 18S ribosomal RNA (18S rRNA) [52] gene for dictyostelids [53] and translation elongation factor 1-a (*EF1-a*) [51,54,55], internal transcribed spacers 1 and 2 within 5.8S rDNA (ITS) [52,56], RNA polymerase II largest subunit (*RPB1*) [51], and RNA polymerase II second largest subunit (*RPB2*) [51] genes for *Morchella* [57] were selected. The primers used for the PCR amplification and sequencing of the five genes are given in Table 1. Each PCR reaction contained 22 μL of T3 Super PCR Mix (Beijing Tsingke Biotech Co., Ltd., Beijing, China), 1 μL of each primer (Sangon Co., Ltd., Shanghai, China), and 1 μL of template DNA; the final volume was 25 μL. PCRs were conducted in a T1000 Thermal Cycle (Bio-Rad, Singapore) using the cycling parameters shown in Table 2. Amplicons were electrophoresed in 1.5% agarose (Sangon Co., Ltd., Shanghai, China) in 1× TAE, stained with Gold View™ (Chongqing Siding Biotech Ltd., Chongqing, China), and then photographed over an ultraviolet transilluminator (Beijing Labgic Technology Co., Ltd., Beijing, China). Then, the PCR products were sequenced with an ABI 3730 capillary sequencer (Sangon Co., Ltd., Shanghai, China). Newly generated sequences were assembled and edited using SeqMan v.7.1.0 (DNA STAR package; DNAStar Inc., Madison, WI, United States). In addition, 54 sequences of *EF1-a*, ITS, *RPB1*, and *RPB2* genes from 28 species previously reported in *Morchella* [57] and 61 sequences of 18S rRNA from 57 species of dictyostelids [53] were retrieved from GenBank and included in the following phylogenetic analysis. Their accession numbers are, respectively, given in Table 3 and Table 4.

Newly generated sequences of dictyostelids and *Morchella* were separately combined in an alignment with downloaded sequences from each genus. In addition, *Lamproderma puncticulatum* and *M. importuna* were chosen, respectively, as the outgroups of dictyostelids and *Morchella*. Sequence alignments were performed separately for each gene dataset with MAFFT v.7.475 using the E-INS-i strategy [58] and then manually checked with BioEdit v.7.0.9 [59]. Maximum likelihood (ML) and Bayesian inference (BI) phylogenetic analyses were conducted for the combined four-gene dataset (ITS-*EF1a*-*RPB1*-*RPB2*) and the 18S rRNA dataset using RAxML v.8.2.12 [60] and MrBayes v.3.2.7a [61], respectively. Rapid bootstrapping with 1000 replicates was executed for ML analysis with the GTR + GAMMA + I model chosen by ModelTest v.3.8 [62]. The BI analysis was run for one million generations, sampling the trees every 100 generations, and used four Markov Chain Monte Carlo (MCMC) chains. When the mean standard deviation of split frequencies was below 0.01, the runs were terminated. The burn-in summary of the top 25% of samples was performed using the “sumt” and “sump” commands to obtain posterior possibilities.

## 3. Results

**Figure 1:**
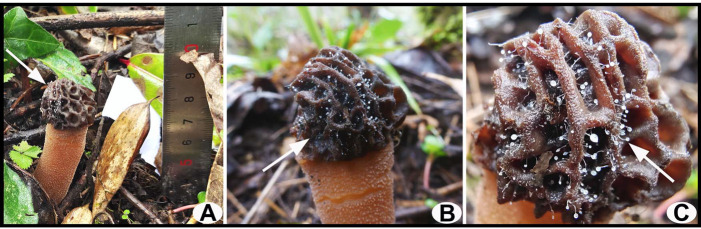
Slime molds colonizing the ascoma of *Morchella* in the field. (**A**) Distant view; (**B**,**C**) close-up view. Slime molds indicated by white arrows.

The substantial proliferation of white and transparent dictyostelids colonizing the cap surface of one *Morchella* ascoma from a forest habitat was found in Chongqing, with the colonized area observed to be blackened and decayed (Figure 1). After a thorough inspection of the surrounding area, only one ascoma with dictyostelids growing on the surface was identified. The specimen of *Morchella* was identified to be *Morchella* sp. *Mel*-21 based on multi-gene phylogenetic analyses. Based on morphological observations, the slime molds were first considered to belong to the genus *Dictyostelium* and were further inferred to be *D. implicatum* by molecular phylogenetic analysis.

### 3.1. Molecular Phylogenetic Analysis of the Morchella Specimen

**Figure 2:**
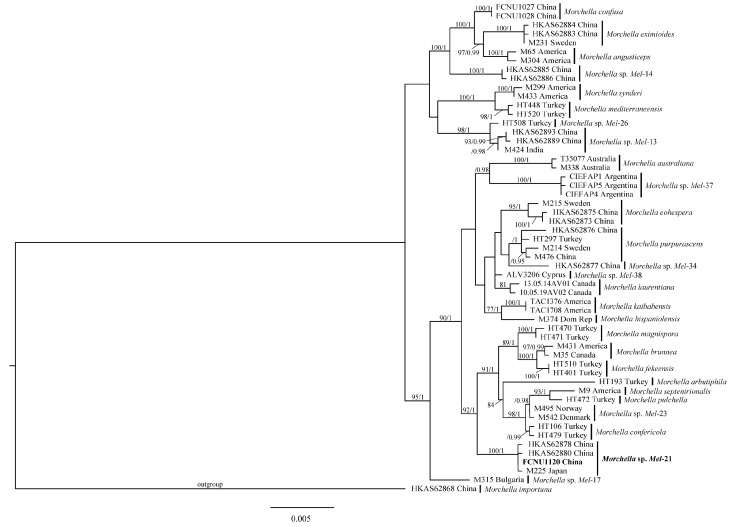
The phylogenetic tree of 28 *Morchella* species inferred from ML analyses based on the concatenated dataset (ITS, *EF1-a*, *RPB1*, and *RPB2*). Bootstrap values over 75% and Bayesian posterior probabilities over 0.95 shown on the branches. The new specimen of *Morchella* used in this study indicated in bold.

In this study, four sequences of the *Morchella* specimen were obtained through PCR amplification targeting the ITS, *EF1-a*, *RPB1*, and *RPB2* genes with accession numbers PP658423, PP695543, PP693901, and PP693900. The alignments of sequences, which included those newly generated in this study and the 54 retrieved sequences from GenBank (Table 3) for ITS, *EF1-a*, *RPB1*, and *RPB2* datasets, respectively, were 646, 777, 692, and 680 bp. The final aligned multi-gene sequence matrix contained 28 species and a total of 55 sequences with 3287 bp. The phylogenetic trees were inferred from the combined four-gene dataset based on ML and BI analyses. No significant topological differences were detected between the two analyses, and the ML phylogenetic tree is shown in Figure 2. The phylogenetic analyses strongly supported the studied specimen being *Morchella* sp. *Mel*-21 (Figure 2) since it clustered together with HKAS62878 and HKAS62880 from China and M225 from Japan with high support (100%/1); these were previously identified as *Morchella* sp. *Mel*-21 [51,63]. Therefore, based on molecular phylogenetic analyses, the species identity of the *Morchella* specimen used in this study was recognized as *Morchella* sp. *Mel*-21.

### 3.2. Morphological Observation of Dictyostelids

***Dictyostelium* sp.**

**Figure 3:**
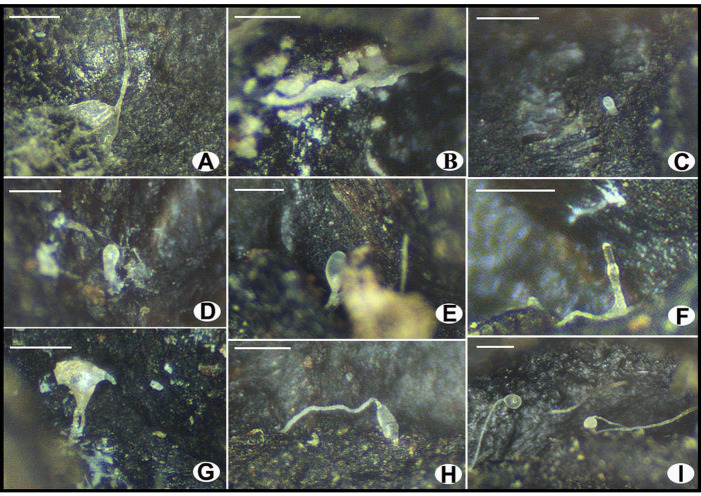
Morphological characteristics of *Dictyostelium* sp. investigated in this study under a stereo microscope. (**A**) Cell aggregation; (**B**) pseudoplasmodium; (**C**–**E**) mexican-hat-like protrusion; (**F**–**H**) the sorocarp formation period with mastoid structure; (**I**) sorocarps. Scale bars = 200 μm.

**Figure 4:**
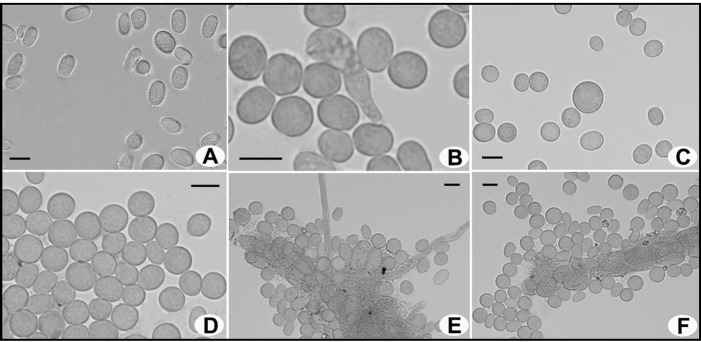
Microscopic morphological characteristics of *Dictyostelium* sp. observed under a light microscope. (**A**) Spores; (**B**) spore germination; (**C**,**D**) microcysts; (**E**) base of sorophores; (**F**) tip of sorophores. Scale bars = 5 μm.

Cell aggregations (Figure 3A) with ample radiate streams. Pseudoplasmodia (Figure 3B) often migrating without sorophore formation. Mexican-hat-like protrusion (Figure 3C–E) and the sorocarp formation period with mastoid structure (Figure 3F–H) observed. Sorocarps (Figure 3I) solitary and unbranched, some erect while others prostrate. Sori white or milk-white, globose. Spores (Figure 4A) hyaline, elliptical, mostly 5.77–7.97 × 3.63–4.85 μm, without polar granules. Spore germination observed (Figure 4B). Microcyst (Figure 4C,D) globose. Sorophores generally stout, tapering from bases to tips, consisting of several tiers of cells, bases (Figure 4E) clavate, tips (Figure 4F) acuminate.

*Specimens examined*. H1054. Isolated from the surface of one wild ascoma of *Morchella* in 2023 from Chongqing, China.

*Known distribution*. China, America, Germany, Korea, Japan, India, Pakistan, Ukraine, Thailand.

*Commentary*. The morphological observation was performed after the samples were dried, and the length and diameter of the fresh dictyostelids’ sorocarps and sori could not be measured. Consequently, the dictyostelids were initially identified as belonging to *Dictyostelium* sp. based solely on morphological features.

### 3.3. Molecular Phylogenetic Analysis of Dictyostelium Implicatum Specimens

**Figure 5:**
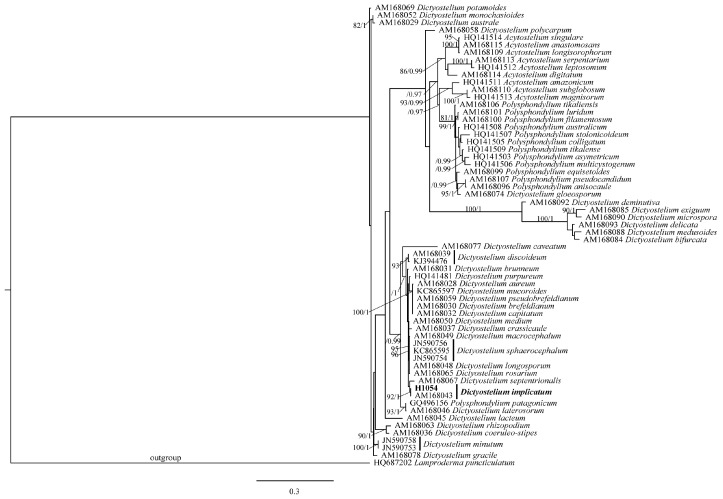
The phylogenetic tree of 57 species of dictyostelids inferred from ML analyses based on 18S rRNA. Bootstrap values over 75% and Bayesian posterior probabilities over 0.95 reported on the branches. The new collection of *Dictyostelium* used in this study indicated in bold.

The newly generated 18S rRNA sequence in this study was 531 bp with accession number PP658424 and aligned with the 61 sequences retrieved from GenBank (Table 4). The final alignment matrix contained 1259 bp with 62 sequences and a total of 57 species. The phylogenetic trees were obtained based on the ML and BI analyses, and the ML tree was presented in Figure 5. The dictyostelids in this study clustered together with AM168043 from Japan with high support (92%/1), which was previously identified as *Dictyostelium implicatum* [64]. Therefore, based on molecular phylogenetic analysis, dictyostelids that colonized the cap surface of *Morchella* ascoma found in this study were identified as *D. implicatum*.

## 4. Discussion

The large-scale commercial cultivation of morels has become a part of an emerging industry for edible fungi in China and globally, showcasing their significant economic and scientific value [6,7]. The colonization of bacteria and fungi is one of the key factors affecting the artificial cultivation of morels and causing serious economic losses [6,21,22,23,24,25]. Increasing numbers of bacteria and fungi, such as *Ps. longispora* [17,18,26,27], *F. incarnatum-equiseti* [16], *C. solani* [21], *Pu. lilacinum* [10], *T. atroviride* [8], *Ps. chlororaphis* subsp. *aureofaciens* and *B. subtilis* [15], *A. alternata* [13], *Pe. raperi* [14], and so on, have been discovered to colonize *Morchella* species and harm their fruiting bodies. Investigating the species diversity of these colonizing organisms is the premise for further revealing their colonization mechanisms and is also crucial for drawing the attention of planters and researchers to them during morel cultivation and in the field.

Dictyostelids, well known as dictyostelid cellular slime molds that feed on bacteria and other microbes [50], have never been reported to act as pathogens of any organisms before [36,37,38,39]. Though tiny and difficult to find in nature with the naked eye [65], dictyostelids have been documented worldwide [46], such as in China, America, Germany, Korea, Japan, India, Pakistan, Ukraine, Thailand, etc. [45,46,47,66,67,68,69,70,71,72,73,74,75,76,77]. In China, they have been previously reported in Beijing, Jilin, Shanxi, Heilongjiang, Liaoning, Hunan, Henan, Xizang, Yunnan, Sichuan, Guizhou, Hainan, Guangxi, Guangdong, Taiwan, and so on [46,47,76,77,78,79,80,81,82,83]. Based on the phylogenetic analysis of ITS, 18S rRNA, 5.8S rRNA, α-tubulin, and β-tubulin genes [64,84,85,86,87], dictyostelids have been reported to include 11 genera [88], of which *Dictyostelium*, *Polysphondylium*, and *Acytostelium* are the most common [89].

In this study, we found that dictyostelids had colonized the surface of one wild ascoma of *Morchella*, with the tissue being black and rotten. The wild ascoma was identified as *Morchella* sp. *Mel*-21 through molecular phylogenetic studies. Interestingly, this species was previously reported to be successfully cultivated in China, albeit with low and unstable yields [90,91]. Though the length and size of fresh sorocarps and sori are crucial for dictyostelids species identification [78], due to the availability of only dried specimens for the microscopic morphological observation of dictyostelids in this study, we were unable to obtain data on their length and size. Consequently, these dictyostelids were initially identified as belonging to the genus *Dictyostelium* based on morphology. Further molecular phylogenetic analysis was conducted to uncover the species identity of the dictyostelids using the 18S rRNA and ITS genes, which are widely accepted for species identification in the genus *Dictyostelium* [89,92,93,94]. However, only a clear 18S rRNA sequence was obtained with clean peaks, while the ITS sequences were always messy after multiple attempts and were then discarded. Based on the phylogenetic tree of the 18S rRNA dataset, the dictyostelids in this study were identified to be *D. implicatum*, with support values being 92%/1, slightly lower than 97%/1, probably due to the newly generated sequence (531 bp) being much shorter than the referenced ones (mainly around 670 bp), chosen according to An and Li [89], but no better sequences of 18S rRNA could be obtained from the dictyostelids after multiple attempts. Notably, to the best of our knowledge, the dictyostelids found in this study are reported for the first time from Chongqing, located in southwestern China, further broadening their distribution record in China.

Considering the lack of previous reports of dictyostelids acting as pathogens of any organisms [36,37,38,39], with the aim of conducting inoculation experiments to determine whether dictyostelids could be pathogens of *Morchella* based on our discovery, we tried to isolate the dictyostelids and hoped to reveal their colonization mechanisms. However, despite multiple attempts across different laboratories, we were unable to successfully isolate them. Given that abundant microbial communities have been reported on the ascomata of *Morchella* [3,4], based on the available literature and our data, we currently hypothesize that the colonization mechanism of dictyostelids discovered in this study is likely consuming the microorganisms present on the surface of the ascoma, and the activity of these microorganisms may, in turn, contribute indirectly to the blackening and decay of the *Morchella* ascoma. Subsequent studies will be conducted to continue trying to resolve this problem and provide a more comprehensive understanding.

This study represents the first report of dictyostelids (*D. implicatum*) colonizing the fruiting body of *Morchella* and introduces novel research advancements for exploring the life history and potential ecological significance of dictyostelids. While organism colonization has previously only been documented in cultivated morels, this study observes it for the first time on wild ascomata. The new finding of dictyostelids colonizing *Morchella* sp. *Mel*-21 could serve as a valuable reference and attract attention for artificial cultivation in the future.

## Figures and Tables

**Table 1:** Detailed information on PCR and sequencing primers.

Locus	Primer	Sequence (5′-3′)	Taxon	Reference
18S rRNA	NS1	GTAGTCATATGCTTGTCTC	*Dictyostelium*	[52]
	NS2	GGCTGCTGGCACCAGACTTGC		
*EF1-a*	EF-595F	CGTGACTTCATCAAGAACATG	*Morchella*	[54]
	EF-1R	GGARGGAAYCATCTTGACGA		[51]
ITS rDNA	ITS1F	CTTGGTCATTTAGAGGAAGTAA	*Morchella*	[56]
	ITS4	TCCTCCGCTTATTGATATGC		[52]
*RPB1*	RPB1B-F	AACCGGTATATCACGTYGGTAT	*Morchella*	[51]
	RPB1B-R	GCCTCRAATTCGTTGACRACGT		
*RPB2*	RPB2B-F	TAGGTAGGTCCCAAGAACACC	*Morchella*	[51]
	RPB2B-R	GATACCATGGCGAACATTCTG		

**Table 2:** PCR programs used for amplification of 18S rRNA, *EF1-a*, ITS, *RPB1*, and *RPB2* in this study.

Gene	PCR Program
18S rRNA	2′ −98 °C, 35× (10″ −98 °C, 10″ −45 °C, 20″ −72 °C), 10′ −72 °C
*EF1-a*	2′ −98 °C, 35× (10″ −98 °C, 10″ −50 °C, 90″ −72 °C), 10′ −72 °C
ITS	2′ −98 °C, 35× (10″ −98 °C, 10″ −50 °C, 20″ −72 °C), 10′ −72 °C
*RPB1*	2′ −98 °C, 35× (10″ −98 °C, 10″ −50 °C, 90″ −72 °C), 10′ −72 °C
*RPB2*	2′ −98 °C, 35× (10″ −98 °C, 10″ −50 °C, 90″ −72 °C), 10′ −72 °C

**Table 3:** Detailed information on the retrieved sequences of dictyostelids used in this study. Newly generated sequence information indicated in bold.

Species	Voucher	Locality	GenBank Accession Number
			18S rRNA
*Acytostelium anastomosans*	PP1	America	AM168115
*A. amazonicum*	HN1B1	Honduras	HQ141511
*A. digitatum*	OH517	America	AM168114
*A. leptosomum*	212rjb	Portugal	HQ141512
*A. longisorophorum*	DB10A	America	AM168109
*A. magnisorum*	08A	America	HQ141513
*A. serpentarium*	SAB3A	America	AM168113
*A. singulare*	FDIB	America	HQ141514
*A. subglobosum*	LB1	America	AM168110
*Dictyostelium aureum*	SL1	America	AM168028
*D. australe*	NZ80B	New Zealand	AM168029
*D. bifurcatum*	UK5	America	AM168084
*D. brefeldianum*	TNS-C-115	Japan	AM168030
*D. brunneum*	WS700	America	AM168031
*D. capitatum*	91HO-50	Japan	AM168032
*D. caveatum*	WS695	America	AM168077
*D. coeruleo-stipes*	CRLC53B	America	AM168036
*D. crassicaule*	93HO-33	Japan	AM168037
*D. delicatum*	TNS-C-226	Japan	AM168093
*D. deminutivum*	MexM19A	America	AM168092
*D. discoideum*	V34	America	AM168039
	M1A	Costa Rica	KJ394476
*D. exiguum*	TNS-C-199	Japan	AM168085
*D. gloeosporum*	TCK52	Japan	AM168074
*D. gracile*	TNS-C-183	Japan	AM168078
*D. implicatum*	93HO-1	Japan	AM168043
	**H1054**	**China**	**PP658424**
*D. lacteum*	/ ^1^	France	AM168045
*D. laterosorum*	AE4	America	AM168046
*D. longosporum*	TNS-C-109	Japan	AM168048
*D. macrocephalum*	B33	Japan	AM168049
*D. medium*	TNS-C-205	Japan	AM168050
*D. medusoides*	OH592	America	AM168088
*D. microsporum*	TNS-C-38	Japan	AM168090
*D. minutum*	Boots_07_A1	America	JN590753
	Boots_07_B1	America	JN590758
*D. monochasioides*	HAG653	Japan	AM168052
*D. mucoroides*	Ice211A1	Sweden	KC865597
*D. polycarpum*	OhioWILDS	America	AM168058
*D. polycephalum*	AP	India	GU562439
*D. potamoides*	FP1A	America	AM168069
*D. pseudobrefeldianum*	91HO-8	Japan	AM168059
*D. purpureum*	cavender	America	HQ141481
*D. rhizopodium*	AusKY-4	Japan	AM168063
*D. rosarium*	M45	America	AM168065
*D. septentrionalis*	AK2	America	AM168067
*D.* *sphaerocephalum*	Ice241A1	America	KC865595
	Boots_14_A2	America	JN590756
	Boots_07_A2	America	JN590754
*Lamproderma puncticulatum*	162	Switzerland	HQ687202
*Polysphondylium anisocaule*	NZ47B	New Zealand	AM168096
*P. asymetricum*	HN20C	Honduras	HQ141503
*P. australicum*	NB1AP	Australia	HQ141508
*P. colligatum*	HN13C1	Honduras	HQ141505
*P. equisetoides*	B7JB	America	AM168099
*P. filamentosum*	SU-1	America	AM168100
*P. luridum*	LR-2	America	AM168101
*P. multicystogenum*	AS2	Africa	HQ141506
*P. patagonicum*	/^1^	Argentina	GQ496156
*P. pseudocandidum*	TNS-C-91	America	AM168107
*P. stolonicoideum*	K12A	Australia	HQ141507
*P. tikalense*	HN1C1	Honduras	HQ141509
*P. tikaliensis*	OH595	America	AM168106

^1^ The voucher information of this sample unavailable.

**Table 4:** Detailed information on the retrieved sequences of *Morchella* used in this study. Newly generated sequences information indicated in bold.

Species	Voucher	Locality	GenBank Accession Number			
			ITS	*EF1-a*	*RPB1*	*RPB2*
*Morchella angusticeps*	M304	America	JQ723055	GU551560	GU551658	GU551707
	M65	America	GU551433	GU551396	GU551470	GU551516
*M.* *arbutiphila*	HT193	Turkey	JN085141	JN085085	JN085201	JN085257
*M. australiana*	M338	Australia	KC753472	KC753468	KC753475	KC753480
	T35077	Australia	KC753470	KC753466	KC753477	KC753478
*M.* *brunnea*	M35	Canada	GU551415	GU551378	GU551452	GU551492
	M431	America	GU551414	GU551377	GU551451	GU551491
*M. confericola*	HT106	Turkey	JN085140	JN085084	JN085200	JN085256
	HT479	Turkey	JN085127	JN085071	JN085187	JN085243
*M. confusa*	FCNU1027	China	MK321848	MK321866	MK321854	MK321860
	FCNU1028	China	MK321849	MK321867	MK321855	MK321861
*M. eohespera*	M215	Sweden	GU551404	GU551367	GU551441	GU551478
	HKAS62873	China	JQ321878	JQ321846	JQ321942	JQ321974
	HKAS62875	China	JQ321890	JQ321858	JQ321954	JQ321986
*M. eximioides*	HKAS62883	China	JQ321898	JQ321866	JQ321962	JQ321994
	HKAS62884	China	JQ321899	JQ321867	JQ321963	JQ321995
	M231	Sweden	GU551428	GU551391	GU551465	GU551508
*M.* *fekeensis*	HT401	Turkey	JN085114	JN085058	JN085174	JN085230
	HT510	Turkey	JN085133	JN085077	JN085193	JN085249
*M. hispaniolensis*	M374	Dominican Republic	MH014725	GU551554	GU551652	GU551484
*M. importuna*	HKAS62868	China	JQ321874	JQ321842	JQ321938	JQ321970
	HKAS62871	Germany	JQ321903	JQ321871	JQ321967	JQ321999
*M. kaibabensis*	TAC-1376	America	MH014727	MH014721	MH014732	MH014737
	TAC-1708	America	MH014728	MH014722	MH014733	MH014738
*M. laurentiana*	10.05.19AV02	Canada	KT819376	KT819387	KT819353	KT819364
	13.05.14AV01	Canada	KT819374	KT819385	KT819351	KT819362
*M.* *magnispora*	HT470	Turkey	JN085122	JN085066	JN085182	JN085238
	HT471	Turkey	JN085123	JN085067	JN085183	JN085239
*M.* *mediterraneensis*	HT448	Turkey	JN085118	JN085062	JN085178	JN085234
	HT520	Turkey	JN085135	JN085079	JN085195	JN085251
*M.* *pulchella*	HT472	Turkey	JN08512	JN085068	JN085184	JN085240
*M.* *purpurascens*	HKAS62876	China	JQ321895	JQ321863	JQ321959	JQ321991
	HT297	Turkey	JN085111	JN085055	JN085171	JN085227
	M214	Sweden	GU551406	GU551369	GU551443	GU551480
	M476	China	GU551426	GU551389	GU551463	GU551505
*M. septentrionalis*	M9	America	JQ723064	GU551556	GU551654	GU551703
*M. synderi*	M299	America	GU551413	GU551376	GU551450	GU551490
	M433	America	GU551425	GU551388	GU551462	GU551503
*Morchella* sp. *Mel*-13	HKAS62889	China	JQ321884	JQ321852	JQ321948	JQ321980
	HKAS62893	China	JQ321888	JQ321856	JQ321952	JQ321984
	M424	India	GU551429	GU551392	GU551466	GU551511
*Morchella* sp. *Mel*-14	HKAS62885	China	JQ321887	JQ321855	JQ321951	JQ321983
	HKAS62886	China	JQ321891	JQ321859	JQ321955	JQ321987
*Morchella* sp. *Mel*-17	M315	Bulgaria	JQ723057	GU551561	GU551659	GU551708
*Morchella* sp. *Mel*-21	HKAS62878	China	JQ321894	JQ321862.	JQ321958	JQ321990
	HKAS62880	China	JQ321882	JQ321850	JQ321946	JQ321978
	M225	Japan	JN085156	GU551559	GU551657	GU551507
	**FCNU1120**	**China**	**PP658423**	**PP695543**	**PP693901**	**PP693900**
*Morchella* sp. *Mel*-23	M495	Norway	JN085153	GU551381	JN085212	GU551495
	M542	Denmark	JQ723063	GU551562	GU551660	GU551709
*Morchella* sp. *Mel*-26	HT508	Turkey	JN085131	JN085075	JN085191	JN085247
*Morchella* sp. *Mel*-34	HKAS62877	China	JQ321896	JQ321864	JQ321960	JQ321992
*Morchella* sp. *Mel*-37	CIEFAP5	Argentina	KJ439678	KJ569626	KJ569594	KJ569620
	CIEFAP71	Argentina	KJ439673	KJ569630	KJ569596	KJ569624
	CIEFAP74	Argentina	KJ439674	KJ569631	KJ569598	KJ569625
*Morchella* sp. *Mel*-38	ALV3206	Cyprus	KU865009	KU865050	KU865040	KU865042

## Data Availability

Newly generated sequences used in the study were uploaded to GenBank with accession numbers PP658423, PP695543, PP693901, PP693900, and PP658424.
